# Bases selection with pseudo-random functions in BB84 scheme

**DOI:** 10.1016/j.heliyon.2023.e23578

**Published:** 2023-12-11

**Authors:** Emir Dervisevic, Miroslav Voznak, Miralem Mehic

**Affiliations:** a Department of Telecommunications, Faculty of Electrical Engineering, University of Sarajevo, Sarajevo, 71000, Bosnia and Herzegovina; b Department of Telecommunications, Faculty of Electrical Engineering and Computer Science, VSB – Technical University of Ostrava, Ostrava, 708 00, Czechia

**Keywords:** Cryptography, Quantum key distribution, BB84, Protocols

## Abstract

Because the spectrum of services available in modern telecommunication networks is constantly expanding, security has become increasingly important. Simultaneously, in an era of constant progress in mathematics and computing, the security of existing cryptographic solutions becomes questionable. Quantum Key Distribution (QKD) is a promising secret key agreement primitive that enables long-awaited practical Information-Theoretical Secure (ITS) communications. The key generation rate, however, is one of the limitations of its widespread application to secure high throughput data flows. This paper addresses the aforementioned limitation by employing perfectly correlated bases selection defined by the output of Pseudo-Random Functions based on the keyed-Hash Message Authentication Code construction. In theory, the proposed variant of the BB84 scheme is ITS, reduces memory requirements, and reduces communication overhead during the post-processing stage. It can benefit QKD networks as a service by increasing capacity and accommodating users with varying security needs.

## 1 Introduction

Many modern digital services necessitate a secure means of communication. This is especially noticeable in the emerging new generation of mobile networks, where the spectrum of digital services is becoming more diverse and includes the manipulation of highly sensitive data. However, as the field of quantum computing continues to advance, it is expected that the current widespread public-key cryptographic solutions will soon become unusable. Without adequate cryptographic alternatives, we can expect our digital lives to be significantly altered and many services to be rendered inoperable [1].

**Figure 1:**
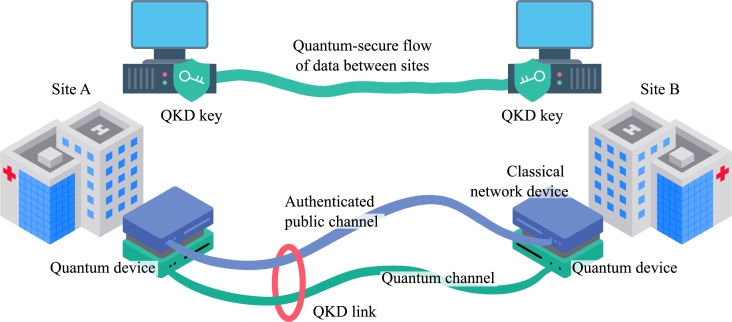
Quantum key distribution.

Quantum Key Distribution (QKD) [2] is a novel cryptographic method based on quantum physics laws that are unaffected by future advances in computing or mathematics. QKD accomplishes one of the most essential and oldest roles of public-key cryptography, namely secret key exchange, in an Information-Theoretical Secure (ITS) manner. This makes possible ITS communications if QKD keys are combined with the One-Time Pad (OTP) cipher in such a fashion that each key is used only once and is as long as the plain text [3], [4]. QKD is a point-to-point technology that allows the exchange of keys between two physically linked parties, as illustrated in Fig. 1. A QKD link is a logical connection formed by a quantum and an authenticated public channel. Random bits are transmitted in non-orthogonal states of quantum systems – particles like photons that unlock unique security features. The authenticated public channel is used to verify and correlate shared information, resulting in symmetric binary sequences known only to the legitimate parties.

**Figure 2:**
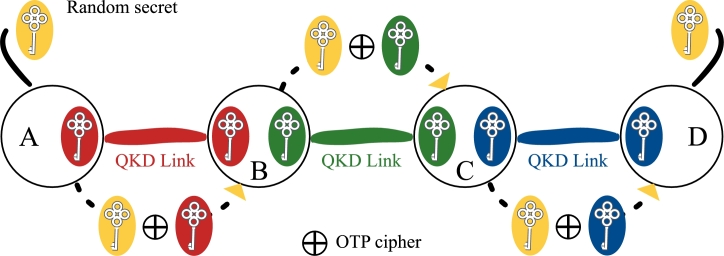
Distribution of the cryptographic key from node A to node D over the QKD network in a hop-by-hop manner. Intermediate nodes must be trusted, for the distributed key to have an ITS security profile. In addition, for the distribution process shown here, the random secret must be truly random, i.e. the output of a quantum random number generator.

For wide-scale QKD application, a network of point-to-point QKD links has been introduced and demonstrated in several testbeds [5]. Based on intermediate trusted-repeater nodes, such networks allow key distribution between any arbitrary network nodes and provide a more robust service than individual QKD links [6]. The key distribution process is illustrated in Fig. 2 as a hop-by-hop process from a trusted node to a trusted node in a connected chain. Networks based on untrusted nodes are also feasible and are commonly deployed as access networks [7], [8], [9]. Due to the high cost of deploying QKD networks for individual organizations and similar entities, the goal is for multiple organizations hosting thousands of users to share the infrastructure of a single QKD network [10]. This is accomplished through the use of sophisticated key and network management methodologies on QKD networks. The primary resource that defines the capabilities of a QKD network as a service is cryptographic keys. The supply of cryptographic keys generated by the QKD process is limited, at best a few Mbps [11], [12], which is low in comparison to data throughput in modern networks. Intelligent key allocation is essential to satisfy the demands of as many users as feasible with restricted network resources [13], [14]. Recent study [15] indicates that in order to offer efficient key resource allocation and high service success probability, client requirements should be relaxed, or investment in the infrastructure layer should be made to increase secret key rates on QKD links. As a result, it appears like an incessant attempt is being made to improve key generation rates [16], [17], [18] while also broadening QKD's reach [19], [20], [21], [22], [23]. Due to the aforementioned limits, QKD's usefulness is confined to low-throughput data flows because it is primarily featured as the key agreement primitive to be utilized with ITS cryptographic techniques. As a result, it is common to feed QKD-derived key material to traditional computationally secure cryptographic algorithms like Advanced Encryption Standard (AES). This broadens the applicability of QKD technology across many critical infrastructures (5G [24], [25], [26], [27], 6G [28], [29], SCADA [30], and smart grids [31], [32], [33]) and allows it to accommodate users with varying security requirements.

This paper proposes a concept that considerably improves the efficiency of the BB84 scheme, the first QKD scheme, thereby shifting the upper bounds of achievable key rates. It has the potential to boost QKD utilization in maintaining security for data flows on high-capacity links by supplying keys at a faster rate. In particular, a variant of the BB84 scheme is proposed in which communication parties use perfectly correlated bases for photon preparation and measurement, which are defined by the output of a Pseudo-Random Functions (PRF) based on keyed-Hash Message Authentication Code (HMAC). The scheme boosts BB84's efficiency from 50 to 100 percent and completely eliminates public announcement of bases during the key establishment process. In theory, the scheme offers ITS profile, and we briefly discuss its security in real-world implementations.

The paper is organized as follows: Section 2 provides a brief overview of QKD and the BB84 protocol. Furthermore, variants of BB84 without public announcement of bases are described, which are closely related to the suggestion made in this paper. Section 3 describes a proposed variant of the BB84 scheme, including a brief discussion of its theoretical and practical security. Our variant of BB84 is then compared with previously researched variants in Section 4. Within the same Section 4, we emphasize the advantages of using our scheme to increase the capacity of QKD networks and accommodate users with varying security needs. Section 5 concludes the study.

## 2 State of the art

This section begins with an overview of QKD and its most well-known protocol, the BB84. Following that, variants of the BB84 protocol that do not require a public announcement of bases are summarized.

### 2.1 Quantum key distribution

The concepts of QKD were established in 1984 [2], when Bennett and Brassard discovered that quantum phenomena could be used to establish a communication channel, i.e., a quantum channel, with prominent security features. These security features, which are a direct result of quantum mechanics rules, prevent adversaries from reliably reading or copying information in transit. As a result, attempts by adversaries to eavesdrop on the quantum channel leave a trace in the transmitted data, revealing their presence to legitimate parties. The secure transmission over the quantum channel, on the other hand, only allows the establishment of correlated, but not symmetric, (partial) secrets between legitimate parties. The authenticated public channel is required to test the correlation, which can reveal eavesdroppers and, in their absence, extract the ITS symmetric keys. The established key is then used within conventional security frameworks (e.g., IPsec [34]) to establish secure communication between the distant parties.

### 2.2 BB84

**Figure 3:**
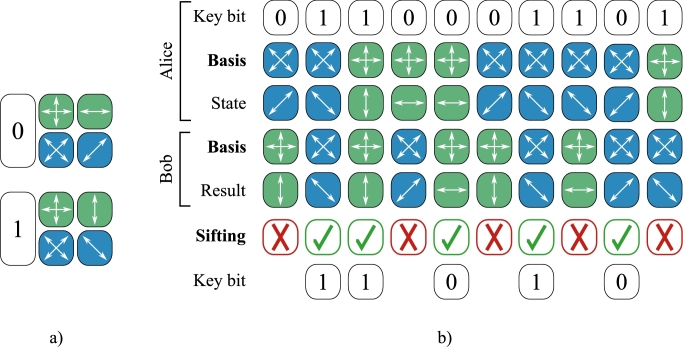
a) A coding dictionary in BB84 scheme. A bit value is encoded in one of two polarization states based on the basis; b) The quantum transfer and the public announcement of bases (sifting) in the BB84 scheme. Because the basis selections of Alice and Bob are independent and random, half of the transmitted information (i.e., the raw key) is discarded, making the basic BB84 scheme 50% efficient.

Bennett and Brassard's original concepts and scheme, proposed in 1984, are now known as the BB84 protocol. The BB84 and its slightly modified variants [35], [36], which improve security in practical implementations, are the most widely used. In the BB84 scheme, the quantum channel transmits single photons with encoded information in their polarization state. To leverage the inclusiveness of quantum measurements, information is encoded in four polarization states that form two conjugate polarization bases, rectilinear and diagonal. Fig. 3a depicts the BB84 coding scheme, and Fig. 3b depicts the starting steps in basic BB84 scheme, where Alice and Bob are legitimate parties involved in the key distribution process.

The BB84 scheme is explained as follows. Alice encodes bits of a random secret in the polarization states of individual photons, with the basis chosen at random. A quantum channel is used to transmit a sequence of single photons from Alice to Bob. Bob measures individual photons in a random basis and remembers the basis and measurement result. When the measurement basis matches Alice's choice of basis, Bob obtains the correct bit value unless an attacker or noise disrupts the transmission. Otherwise, as the laws of quantum mechanics indicate, the outcome is entirely random.

Once a quantum transfer, or communication over the quantum channel, is complete, Alice and Bob proceed to align the shared, correlated bits, which are called the raw keys. To avoid a man-in-the-middle attack, all subsequent communication between Alice and Bob takes place over an authenticated public channel. The first step that follows the quantum transfer is a public announcement of bases, also known as the sifting phase, in which Bob publicly announces his measurement bases, and Alice informs him which measurements were correct. They then retain the portion of the raw key where the bases match. Because Alice and Bob's bases selections are *random* (with equal probability ($\frac{1}{2}$) of occurrence) and completely *independent* of one another, the probability that they choose the same bases is given by Equation (1), where A and B are two bases, rectilinear and diagonal, respectively. Therefore, the efficiency, i.e., protocol gain of the BB84 is 50% ($g_{p}=0.5$).

(1) $$P(A\cap A)+P(B\cap B)=P(A)\cdot P(A)+P(B)\cdot P(B)=\frac{1}{2}\cdot \frac{1}{2}+\frac{1}{2}\cdot \frac{1}{2}=\frac{1}{2}$$

Following the public announcement of bases, Alice and Bob perform error estimation and reconciliation [37], [38], [39], as well as privacy amplification [40] steps. After their public discussion, Alice and Bob successfully rendezvous a secret symmetric key.

### 2.3 BB84 without public announcement of bases

As a result of Alice and Bob's uncorrelated, random choice of bases, half of the raw key bits are discarded at the public announcement of bases (see equation (1)). Alice and Bob may always agree to utilize one of the bases with higher probability *δ* ($\frac{1}{2}<\delta <1$), resulting in increased BB84 efficiency [41]. This scheme is known as the asymmetric BB84 protocol, and its efficiency can be made asymptotically close to 100%. Equation (2) gives the protocol gain [42].

(2) $$g_{p}=P(A\cap A)+P(B\cap B)=P(A)\cdot P(A)+P(B)\cdot P(B)=\delta \cdot \delta +(1-\delta )\cdot (1-\delta )=2\delta^{2}-2\delta +1$$

When the number of transmitted photons $m_{t}$ approaches infinity ($m_{t}\to \infty$), *δ* can approach the value of 1 (δ→1), implying that the protocol's efficiency approaches 100% ($g_{p}\to 1$). However, on a finite set of transmitted photons (as is the case in practice), *δ* cannot take on value arbitrary close to 1, because the number of photons prepared and measured in a basis with a low probability of occurrence (1−δ) would be insufficient to make an accurate estimate of error and detect the eavesdropper.

On the other hand, there are less known variants in which Alice and Bob's basis selections are perfectly correlated, eliminating the need for public announcement of bases (note that the asymmetric BB84 still requires this step). BB84's efficiency is maximized in this manner ($g_{p}=1$). These variants are described in the following paragraphs. The emphasis is on the fundamental approaches, with little thought given to the security of the variants under consideration.

**Figure 4:**
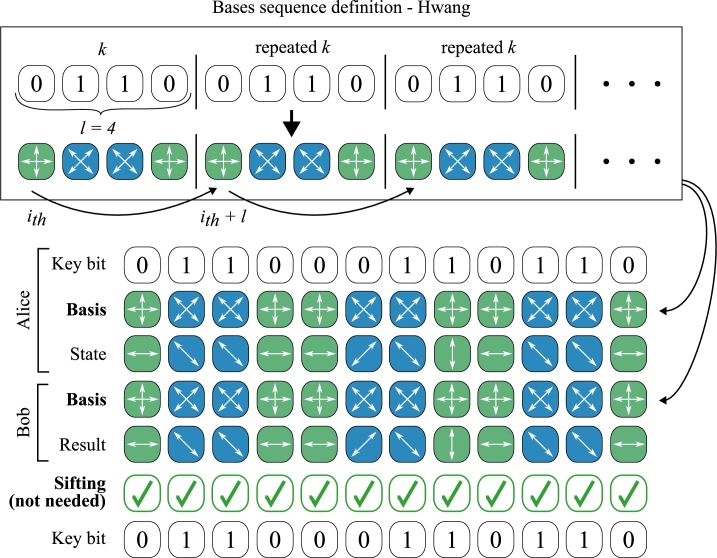
The basic concept underlying the Hwang protocol. Figure shows a smaller-scale example where the pre-shared secret *k* is *l* = 4 bits long. Due to perfectly correlated bases selections the protocol efficiency is 100%.

To the best of our knowledge, the Hwang protocol [43], illustrated in Fig. 4, is the first variant of the BB84 scheme that does not require public announcement of bases. A perfectly correlated bases sequence *k* must be known a priori to legitimate parties and is suggested to be established using the basic BB84 scheme. The scheme is only effective if the bases sequence can be safely reused multiple times; otherwise, the resulting keys would be entirely used to define a new bases sequence. Convenient enough, the authors suggest that bases sequence can be safely reused because the eavesdropper, even knowing which of the quantum carriers, i.e., single photons carrying the information, are encoded in the same basis, cannot determine the basis itself. In theory, the Hwang protocol is proven to achieve ITS security profile [44].

In [45], a Quantum Key Expansion (QKE) scheme based on a varied version of Hwang protocol is introduced. A common preshared secret key, whose length is required to be twice the length, donated as *N*, of the secret key being distributed, defines the polarization state in which single photons are prepared and the encoding operation which generate transformation of the eigenstates within the basis. A newly distributed key of length *N* is reconciled (privacy amplification is optional) and merged with a preshared key that has been privacy amplified.^1^ The scheme works as long as the merged key size is greater than *2N*. Additionally, the authors have proposed a higher-dimension extension of their scheme, which significantly improves security and demonstrates that the maximum distance for secure key distribution can be greatly increased compared to the basic BB84 scheme.

In [46], a floating basis protocol is introduced. In the suggested protocol, a possible number of bases in a single-dimensional Hilbert space is infinite.^2^ To improve protocol's characteristics, Alice and Bob share a secret key a priori (referred to as an auxiliary key) that allows them to correlate their choice of bases. In addition to the maximum efficiency, the benefits of this protocol are as follows: The eavesdropper's trace is more visible (i.e., the eavesdropper introduces more errors), the eavesdropper's knowledge of the secret key is diminished, and the threshold for Quantum Bit Error Rate (QBER) corresponding to the secure transmission increases. The protocol and its security are discussed in [47], while the combination of the floating bases and decoy states is presented in [48].

A protocol with pseudo-random choice of bases (PRB) has been proposed in [49] as a formalization of the floating basis protocol. The pseudo-random sequence, generated by a Legendre symbol Pseudo-Random Number Generator (PRNG), determines the rotations by an arbitrary angle (from a finite set) of the standard basis (i.e., rectilinear). A small secret, known a priori, is used as a seed to the PRNG. The authors showed that the multi-bases variant of the suggested scheme outperforms the BB84 and the asymmetric BB84 protocol regarding the secret key rates. However, the protocol crucially requires single-photon sources. Otherwise, the eavesdropper could guess the initial secret (and thus, the pseudo-random bases) with a non-negligible probability of intercepting only a small number of three-photon pulses using the Photon Number Splitting (PNS) attack [50].

Similarly, the authors of [51] suggest that the secret key be used as a seed in the PRNG, resulting in a so-called running key that defines the bases sequence. The authors of [52] propose a variant of the Hwang protocol in which the base sequence is defined in a pseudo-random manner using cipher block chaining. A priori, Alice and Bob must share two secrets: the bases sequence and the initialization vector required in the cipher block chaining algorithm. Furthermore, a family of coherent-state quantum key distribution protocols with correlated pseudo-random bases sequence has been introduced in [53], [54].

Most recently, in [55] improved variant of the Hwang protocol has been proposed in which shift register, filled with secret bits, is used to define the basis sequence. Assuming the key distribution technique yields a secret key of length *m*, the content of the register is shifted to the left by *m* bits, and the distributed secret key is appended to the shift register. In this manner, a bases sequence is updated for each protocol's round. The leftmost bits pushed out of the register due to the shift are passed through the key derivation function based on universal hash functions. The outcome provides key that is used for cryptographic purposes.

## 3 Bases selection with PRFs based on HMAC construction

Assuming that Alice and Bob share an ITS secret, they can utilize it to define a significantly larger secret in the traditional manner. Because the expanded form is not truly random (true randomness of expanded form can only be achieved through the proven process of QKD), it would be naive and incorrect to use it as an ITS secret key in any cryptographic task. If, on the other hand, the secret or its expanded form is never revealed, the adversary has a negligible chance of guessing it. This section describes a specific method for expanding the secret in a traditional manner, as well as the benefits obtained when such expanded form is used as a definition of correlated bases selection in the BB84 scheme.

### 3.1 Bases selection

In the proposed scheme, Alice and Bob use a small amount of preshared key material obtained from the basic BB84 scheme or the previous execution of the scheme proposed here to create a (larger) shared secret that defines bases selection. By concentrating the outputs of the PRFs based on the HMAC construction, the bases sequence is defined by Equation (3), where *K* and *S* are the ITS symmetric keys known a priori to the communication parties, 0x01, 0x02, etc. are single octets,^3^ and a symbol | represents a concentration. In general, an input message *S* does not need to be secret; however, we argue that a secret message *S* provides additional security.

(3) basessequence=T1|T2|T3|T4|...T1=PRF(K,S|0x01)T2=PRF(K,T1|S|0x02)T3=PRF(K,T2|S|0x03)T4=PRF(K,T3|S|0x04)...

**Figure 5:**
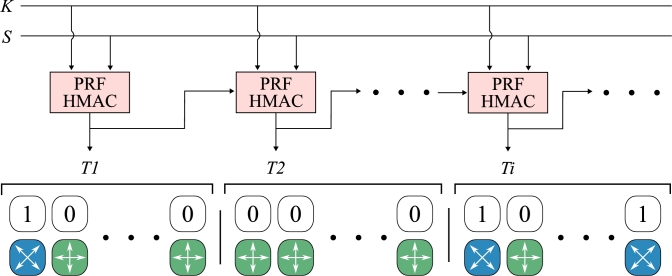
The general idea of bases selection with PRF based on HMAC construction; *K* and *S* are ITS secrets established with basic BB84 scheme or previous execution of scheme proposed here; *Ti* are outputs of PRF functions based on HMAC construction and thus, are uniform in distribution and indistinguishable from a true-random sequence. This figure does not depict additional octet inputs to PRFs.

Fig. 5 depicts this procedure, which is none other than HMAC-based extract–and–expand Key Derivation Function (HKDF) [56]. However, only the second step – expand – is used in this case, with the first step – extract – skipped. Randomness extractors (which may or may not be based on HMAC) are used to extract highly random output with a uniform distribution from a weak random entropy source [57]. In our case, the secret key *K* is a true–random sequence, so the randomness extractor is not applied. It should be noted that for each QKD protocol round, *K* and *S* are refreshed.

The key consumption during the bases selection process (3) is minuscule (e.g., if HMAC SHA-512 is used as a PRF, the key *K* must be 512 bits in size, and the secret message *S* can be as short as 128 bits) in comparison to the efficiency benefits provided by the scheme, i.e., roughly doubling the final key size/rate of the basic BB84 scheme. The final key rate is directly proportional to the sifted key rate, which can be defined in a simplified form by the Equation (4), where *ν* is source repetition rate, *μ* is mean photon number (μ≃0.1),^4^ $T_{link}$ is attenuation of the fiber, i.e., a probability of photon to arrive the detection suite, and *η* is detection efficiency [58], [59].

(4) $$R_{sifted}=g_{p}\cdot R_{raw}=g_{p}\cdot (\nu \cdot \mu \cdot T_{link}\cdot \eta )$$

Because the bases selections are perfectly correlated, our proposed scheme has a 100% efficiency factor ($g_{p}=1$), which is double the 50% efficiency factor of the basic BB84 scheme. As a result, the sifted key rate (4) is also doubled as shown by Equation (5).

(5) $$\frac{g_{p-proposed}\cdot R_{raw}}{g_{p-bb84}\cdot R_{raw}}=\frac{1\cdot R_{raw}}{0.5\cdot R_{raw}}=2$$

### 3.2 Security considerations

To jeopardize the proposed scheme's security, the eavesdropper would have to break the (3) by disclosing *K* and *S*. However, this appears to be impossible without knowing the bases sequence itself (how can one disclose inputs of HMAC, without knowing the output?). The laws of quantum mechanics prevent the eavesdropper from gaining any knowledge on the bases of the intercepted quantum carriers. The eavesdropper can intercept and measure quantum carriers; however, the result does not reveal any information about the basis, and the eavesdropper cannot be certain that the measurement was compliant. As a result, because the eavesdropper knows nothing about *K*, *S*, and outputs *Ti*, there is no attack strategy to reveal the bases sequence. To the eavesdropper, the bases sequence appears as a true-random sequence. Because it is a slight variation of the Hwang protocol, the exact security proofs apply, and thus the proposed scheme is, in theory, ITS secure [44].

Furthermore, because the proposed scheme does not require public announcement of bases at all, the eavesdropper uncertainty is not alleviated, and the amount of leaked information under incoherent attacks is lower when compared to the basic BB84 scheme [43]. As a result, the secret key rate of the proposed scheme *S* is no worse than that of the BB84 scheme, and is given with Equation (6), where I(X;Y) is a mutual information shared by Alice and Bob, I(X;Z) is a mutual information shared by Alice and the eavesdropper, i.e., the amount of leaked information, *h* is a binary entropy, and *e* is the QBER [59].

(6) $$S\geq S_{BB84}=I(X;Y)-I(X;Z)=1-h(e)-2\cdot e$$

The equation (6) applies to a full intercept and resend attack in which the amount of information shared by Alice and the eavesdropper is $I(X;Z)=2\cdot e=\frac{1}{2}$ [42].^5^ In this attack, the eavesdropper listens to the public announcement of bases to eliminate measurement uncertainties. Because our protocol does not require public announcement of bases, neither the full intercept and resend attack nor the intercept and resend attack in the Breidbart basis apply. As a possible strategy, the eavesdropper is left with a naive intercept and resend attack. In this case, the amount of information leaked is significantly less I(X;Z)≃0.2 [42] and the secret key rate is $S=1-h(e)-\frac{4}{5}\cdot e$.

For the purposes of discussing a practical security, let's assume that the eavesdropper has partial information about the bases sequence defined by (3). The output of the PRFs based on HMAC construction is uniform in distribution and indistinguishable from random. We argue that it is challenging to reveal secrets *K* and *S* with only partial knowledge of the PRFs' outputs $T_{i}$. The fact that only a small percentage of pulses are non-empty (μ≈0.1), and only about 5% of them contain more than one photon [58], means that the eavesdropper can determine only a few bits of the output *Ti* using PNS attack.^6^ The cryptographic hash functions (which are an integral part of HMAC), have a one-way, or pre-image resistance property, which means that given the output H(x) and the hash function *H*, it is still computationally infeasible to find the input *x* [60]. But given only a fraction of the output H(x) (as in the proposed application), finding the input *x* would certainly be much more difficult. In general, most hash function attacks assume knowledge of the output (and, in some cases, the input) and hash function, but in the proposed application for bases selections, only a fraction of the output (thus, the input for the following $T_{i+1}$ calculation) can be known. The computational security of the one-way property, or other properties of the underlying hash function, raises concerns. However, even the large-scale quantum computing is expected to weaken rather than break the security of hash functions [1]. As a result, the use of quantum-resistant hash functions (e.g., SHA-2, SHAKE, SHA-3, RIPEMD, Blake2 [1]), is required, preferably in 256 bit and higher variants. It should be noted that a successful attack on (3) is only beneficial for a short period of the quantum transfer, and if this short-term security can be guaranteed, the security of distributed key can also be guaranteed under that assumption.^7^ If a hash function is broken in the future, it will not affect the security of previously established keys or sensitive data protected by them. The scheme can then be easily modified by implementing a new quantum-resistant hash function. This is not the case with other computationally secure key distribution protocols, in which key exchange and confidential data can be recorded, cracked, and reviled after the fact. To improve security in practical implementations, a scheme can be combined with decoy states, primarily to detect passive eavesdropping on multi-photon pulses. However, we do not provide quantitative amounts of security in practical realizations in this paper, instead focusing on the possibilities that the proposed method would provide in light of current trends in QKD networks. We discuss this in Section 4, where we share the light on how our protocol compares to others in some practical sense, as well as the benefits of using our proposed method.

Furthermore, because Alice and Bob use perfectly correlated bases selections, a multi-base variant of the proposed scheme is feasible without sacrificing efficiency. In this case, the proposed scheme can be viewed as a formalization of the floating basis protocol (see Section 2.3), which includes all the advantages of this protocol.

## 4 Discussion

This paper presents a variant of the BB84 scheme that does not require public discussion of base selections. In theory, the proposed variant is ITS. This security is inherited from the base Hwang protocol, whose security has been proven. However, the question arises as to how to benefit from these QKD schemes, whose security in practical applications is not known. It is justifiable to conclude that some of the suggested solutions do not work in practical environments because they fail to take into account a drawback of realistic single-photon sources (i.e., faint laser pulses with very low mean photon number *μ*): most of the pulses are empty [58]. This is why the Hwang protocol would necessitate numerous repetitions of a basis sequence *k* to accumulate sufficient raw key material, or the pre-shared secret *k* would be impractically large. Similarly, source and medium capabilities are disregarded in solutions that assume that a *N* bit base sequence is sufficient to provide an equal number of raw/sifted key bits.^8^ Therefore, these solutions cannot be implemented in practice without reusing, i.e., repeating, the base sequence. Significant correlation may threaten security in a real-world setting by simply reusing the bases sequence, as in the Hwang protocol. This is because of an additional drawback where light pulses in practical single-photon sources may contain more than one photon [58]. Using the PNS attack on the Hwang protocol, after 50 reuses, one can obtain all of the basis's information without being detected [55]. To overcome these limitations, the basis sequence cannot be arbitrarily long, since this would result in a protocol that uses more secret key material than it produces. The most recent method, based on a shift register [55], is an exception. The memory requirements, however, would be substantial, and there would still be a significant correlation between succeeding generation repetitions. This is because a considerable amount of the base sequence remains unchanged and is simply sifted by the length of the newly generated key. During the key distillation process, an adversary can discover the length of the generated key and thus the sift. As a result, the identical problem revealed in the Hwang protocol is present here. Our scheme, on the other hand, does not have these limitations and does not require the storage of base sequences other than the two outputs (of relatively small size, 256, 512 bits, or larger, depending on the hash function used), $T_{i-1}$ and $T_{i}$ at the time. The quantity of key material to keep the scheme functioning, i.e., key consumption, is minimal.

Compared to more practical variants [49], [51], [52], we argue that PRFs based on HMAC construction are more secure than a simple PRNG or one based on cipher block chaining. It is no accident that this method is the most often used to generate several cryptographic keys from a single secret. However, quantitative security analysis is still lacking in our proposal. Compared to the asymmetric BB84 protocol, which has relatively large memory requirements, our method requires fewer resources. This is due to the fact that in order to provide the same level of security as the standard BB84 protocol, the asymmetric BB84 protocol needs a significantly higher quantity of raw key material. This increases the load since successive key generation instances need a longer time frame, especially if one of the rounds fails due to inordinate noise. This may affect achievable key rates in continuous operation in practical deployments.

Compared with traditional key exchange methods, the method proposed here has considerable security advantages, even in the case of imperfect practical technology. Classical key exchange algorithms that are commonly used, as well as potential post-quantum ones, may be simply captured with the encrypted data they protect. If the algorithm has been broken or weakened by technological advancements, the key is revealed along with the encrypted data. This “store now and decrypt later” attack does not apply to the method described here. An attack on our key exchange method is only effective while it is being executed. Breaking the HKDF algorithm after the fact has no effect on previously established secret keys and does not put previously transmitted confidential data at risk. As a result, as long as we can affirm with certainty that HKDF is secure during a quantum transfer, the established key can be used with the OTP cipher to provide long-term security to confidential data. This is a novel middle ground between QKD's overly strict ITS security profile and the dubious long-term security of post-quantum algorithms. The QKD network is anticipated to support an extensive user base with a range of security requirements. As a result, users that depend on computationally secure algorithms, such as AES, only require to have a key that is at least as secure as the encryption algorithm itself, rather than an ITS cryptographic key. We argue that the scheme proposed here is more secure (in practical applications, using imperfect quantum technologies; in theory it is proven to be ITS) than classical and post-quantum key exchange methods, and can provide secret keys with everlasting security based on guaranteed short-term security. It should be recognized as such and utilized within present QKD networks to supply keys at a faster rate, drawing greater attention as a viable key exchange primitive. The advantage is that we can simply switch between the standard BB84 protocol and the one presented here, allowing us to serve customers with various security needs.

## 5 Conclusion

In this study, we propose that PRFs based on the HMAC construction be used to define the perfectly correlated selection of bases in the BB84 scheme, eliminating the need for the public announcement of bases. This concept not only leads to increased scheme efficiency (from BB84's original 50% to a 100%) and key rates, but it also allows for significant additional benefits in multi-bases variants. The scheme requires only a small amount of preshared ITS bits (in each QKD protocol round) to operate, which may be obtained from the basic BB84 scheme or the previous execution of the proposed scheme. The proposed scheme is, in theory, ITS secure. Quantitative amounts of security in practical deployments are lacking and have yet to be provided. The scheme has the potential to expand the capacity of QKD networks, allowing them to serve more users while meeting their diverse security requirements.

## CRediT authorship contribution statement

**Emir Dervisevic:** Writing – review & editing, Writing – original draft, Visualization, Validation, Methodology, Investigation, Formal analysis, Data curation, Conceptualization. **Miroslav Voznak:** Funding acquisition, Formal analysis. **Miralem Mehic:** Supervision, Formal analysis.

## Declaration of Competing Interest

The authors declare that they have no known competing financial interests or personal relationships that could have appeared to influence the work reported in this paper.

## Data Availability

No data was used for the research described in the article.

## Footnotes

1 The authors suggest that there is no way for an eavesdropper to obtain information about the distributed key, so privacy amplification is optional. However, the eavesdropper may obtain partial information about the preshared key, necessitating privacy amplification prior to the merging operation.

2 In theory, there are an infinite number of possible bases, but in practice, this number is limited by the ability to represent basis position accurately.

3 Instead of a single octet, two or more octets may be used to allow for greater expansion, i.e., calculation of many *Ti* outputs without restarting the octet counter.

4 Due to the lack of perfect single photon sources, practical implementations rely on faint laser pulses with very low mean photon number [58].

5 If the eavesdropping applies to all quantum carriers, the amount of errors *e* introduced by the eavesdropper in the BB84 scheme is $e=\frac{1}{4}$. If however, only a fraction $p_{IR}$ of quantum carriers are intercepted, the QBER is defined as $e=\frac{p_{IR}}{4}$ and the amount of leaked information is given as $I(X;Z)=\frac{p_{IR}}{2}=2\cdot e$.

6 If SHA-512 is considered as PRF, the PNS attack can only yield about 2 to 3 bits on given $T_{i}$ on average.

7 It should be noted that similar assumptions are sometimes made for public channel authentication, where instead of ITS authentication, parties choose to use quantum-resistant schemes. This decision is most likely to be made during the initial execution of the QKD process, when the parties have not previously shared any ITS key material. The subsequent rounds of the QKD process may use ITS authentication with previously established keys.

8 Losses in the transmission medium might prevent light particles from reaching the detection suite.
